# Exploring gender influence on adolescent awareness and perspectives regarding social, economic and environmental sustainability

**DOI:** 10.1186/s12889-025-24046-8

**Published:** 2025-08-14

**Authors:** Salvador Boned-Gómez, Alberto Ferriz-Valero, Ove Østerlie, Salvador Baena-Morales

**Affiliations:** 1 Faculty of Education, Centre for Higher Education Alberta Gimenez (CESAG), Comillas Pontifical University, Madrid, Spain; 2 Faculty of Education, General Didactics and Specific Didactics, University of Alicante, Alicante, Spain; 3 Faculty of Social and Educational Science, Department of Teacher Education, NTNU – Norwegian University of Science and Technology, Trondheim, Norway; 4 General Didactics and Specific Didactics, Faculty of Education, University of Alicante, Alicante, Spain

**Keywords:** Public health, Education, Youth engagement, Environmental literacy, Consciousness

## Abstract

**Background:**

This study investigates gender differences in sustainability awareness among adolescents, focusing on how these differences may influence education for sustainable development (SD). Based on an analysis of existing literature, which presents varied perspectives on the impact of gender on sustainability awareness, this work seeks to address a significant gap in the current knowledge.

**Methods:**

A cross-sectional quantitative study was carried out on adolescents in the province of Alicante (Spain) through the completion of the SCQ-S questionnaire in the period from March 2022 to May 2023.

**Results:**

The results reveal higher sustainability awareness among female adolescents compared to males, except for in one dimension where no significant differences were detected. Employing a cross-sectional questionnaire-based study conducted in person as the methodology, with 1,192 participants (48.33% male and 51.67% female), the SQS-S questionnaire was used to measure awareness about sustainability.

**Conclusions:**

This study not only contributes to the understanding of how gender equality can be integrated into educational systems, in line with SDGs 4 and 5, to promote SD, but also proposes the establishment of benchmark scores based on percentiles. These scores will enable the assessment of sustainability awareness levels among adolescents, thereby offering educators a tool to accurately identify the specific needs of their students. We argue that educational interventions must be meticulously designed to be inclusive and sensitive to gender differences, thereby enhancing sustainability awareness in all students and contributing to the development of a more equitable and sustainable society. This body of research underscores the importance of adopting a holistic approach to sustainability education, recognising the crucial role of the educational environment in supporting this learning and the adoption of sustainable behaviours.

## Introduction

Sustainability awareness is crucial for the future well-being of our planet, and needs to be cultivated from an early age and throughout a person’s formative period [1, 2]. In this regard, education for sustainable development (ESD) emerges as an essential tool for fostering this awareness, with its primary goal being to identify ways in which the educational system can be as effective as possible in developing citizens who are committed to the most relevant sustainability issues [3]. The United Nations 2030 Agenda and its Sustainable Development Goals (SDGs) represent an ambitious global action plan that is aimed at addressing some of humanity’s most pressing challenges, from eradicating poverty to combating climate change and promoting gender equality [4]. It is here that the need arises to address and analyse these global goals through more concrete and detailed prisms [5]. The specific case of gender equality, which is primarily framed under SDG 5, has been the subject of numerous studies and evaluations, focusing largely on its economic and social dimensions [6, 7]. However, the role of gender equality from an environmental perspective also deserves deep attention and analysis [8, 9]. This holistic approach, which considers its implications on environmental sustainability in addition to social and economic aspects, opens new avenues for more inclusive and effective research and policies. Therefore, gender equality has been posited as one of the fundamental SDGs that articulates SD [10].

### Education, gender and sustainability

Within the educational sphere, it is recognised as an essential practical approach – not just as an academic goal – to foster inclusive societies and promote better public health outcomes [3, 11]. The existing literature has begun to explore gender perspectives in relation to the right to education and disparities in educational opportunities, which contributes to an emerging understanding of the effectiveness of educational interventions that are sensitive to gender differences [12]. Furthermore, research on strategies to achieve gender equity in educational settings, especially in the context of sustainability, is identified as crucial to shed light on the broader implications for health and the social benefits of an education that is sensitive to gender differences [13].

Within this context, there is a clear need for studies that provide empirical insights and practical recommendations to promote gender equality and awareness of sustainability among youths [14]. A deeper analysis of potential differences in sustainability awareness between genders can support the creation of more equitable and healthy educational environments, aligning with global efforts to understand and foster educational practices that promote engagement with SDG 5 [15]. Therefore, it appears to be important to integrate gender-related issues into the classroom to eliminate bias and discrimination, thereby achieving a comfortable and conducive learning environment for all students [16]. Furthermore, it has been identified that both gender equality and women’s rights are intrinsically linked to at least 11 of the 17 SDGs, which underscores the need for reliable indicators related to gender dynamics for progress towards these goals [17]. Friedman et al. [18] highlight that, although globally we are on track to achieve almost universal primary education by 2030, substantial challenges remain in completion rates for secondary and tertiary education.

In this regard, during adolescence, young people begin to forge their own perceptions of the world and develop attitudes and behaviours that will influence their interaction with the social and natural environment [19]. Developmentally, adolescents gradually acquire the ability to think abstractly and critically, as described in Piaget’s theory of cognitive development [20]. This cognitive maturation, combined with progression through Kohlberg’s stages of moral reasoning [21], may influence how young people perceive and prioritise sustainability issues. In addition, it has recently been discovered that during adolescence the prefrontal cortex of the brain remodels [22]. This is very relevant because this part of the brain is related to decision-making and is responsible for the ability to plan and consider the consequences of actions, as well as to control impulses. In this context, education emerges as an essential pillar for the promotion of sustainability and gender equality, offering a unique opportunity to positively impact the development of adolescents and, thereby, the future of our societies [23]. Therefore, effective ESD should focus on fostering a critical awareness of sustainability among young people, as well as the skills for making responsible decisions and actively participating in the construction of a sustainable future [24]. However, research has shown that there are gender gaps in sustainability awareness among students, which suggests that girls may have a greater sensitivity towards these issues compared to boys [25]. Moreover, exposure to role models who challenge traditional gender stereotypes can play a crucial role in expanding the personal and professional aspirations of adolescents, especially girls, in fields that are less traditional for their gender, such as science, technology, engineering and mathematics (STEM) [26].

#### Does gender influence awareness of sustainable development among adolescents?

The answer to such a broad question is not straightforward. A study conducted in Sweden by Olsson and Gericke [25] revealed a significant gender gap in sustainability awareness among students aged 12 to 19 years. This finding is particularly relevant as it indicates that girls exhibit a higher awareness of sustainability issues compared to boys, and that this gap widens as the students age. Additionally, Lane et al. [27] highlight early adolescence as a crucial period for the development of equitable attitudes and norms between genders, which are intrinsically linked to sustainability awareness. Olsson et al. [28] complement this perspective by investigating the “decline” in sustainability awareness during adolescence, suggesting that ESD could be a valuable tool for addressing and reversing this trend.

However, recent studies suggest that the relationship between gender and sustainability awareness may be more complex and nuanced, indicating that structural, organisational and cultural factors play a critical role in this dynamic. Kassinis et al. [29] provided evidence that organisational policies and gender diversity on boards are significantly related to the adoption of environmentally sustainable practices. In a different context, Lee [30] explored green purchasing attitudes and behaviours among adolescents, and found that whilst female adolescents showed greater environmental concern, male adolescents exhibited a higher level of self-identification with environmental protection. In line with these findings, Calabrese et al. [31] analysed gender differences in expectations and perceptions of corporate social responsibility, and discovered that, although there were minor differences in expectations, perceptions of this social responsibility did not show significant differences between genders. Finally, Alarab et al. [32] investigated the level of awareness among young people about gender equality, and concluded that efforts to promote it are effectively reaching both genders. Therefore, the evidence suggests that these differences are complex and influenced by a variety of structural, organisational and cultural factors. As observed, the current scientific literature presents a diverse array of perspectives on the influence of gender on sustainability awareness [25, 32, 33], which indicates a lack of consensus and comprehensive understanding of how both genders perceive and engage with the three dimensions of SD.

Given the inconsistency in the previous literature, the present study proposes an analysis of the levels of awareness about sustainability – specifically in terms of knowledge, attitudes and behaviours – in the social, economic and environmental dimensions of SD among adolescents, with a particular focus on the influence of gender. Secondly, it aims to establish benchmark scores, defined in percentiles, to measure levels of sustainability awareness in adolescents. These scores will serve to help educators to understand how their students stand in relation to behaviours, attitudes and knowledge about SD. Based on the aforementioned objectives, the following hypotheses are proposed to guide the study: (H1) There are significant differences in the levels of knowledge, attitudes and behaviours related to sustainability between genders, which can be observed through the social, economic and environmental dimensions of SD. (H2) The implementation of percentile-based benchmark scores will reveal significant variations in sustainability awareness among students, allowing for the identification of specific areas of strength and opportunities for improvement in relation to SDGs. Based on the results obtained, recommendations can be proposed for the design of future educational strategies that, framed in gender equality, promote true development and commitment to sustainability needs.

## Methodology

### Participants

A quantitative cross-sectional design was used for this study. A total of 1,262 questionnaires were collected, of which 70 were discarded due to deficiencies in their completion. Therefore, 1,192 adolescents (with an average age of 17.2 ± 2.5 years; 51.67% females) were ultimately included in the study. All participants were students from various educational centres in the province of Alicante, Spain. Regarding academic level, the students were distributed across different grades: first, second, third, and fourth year of compulsory secondary education (ESO), as well as first and second year of Bachillerato (non-compulsory secondary education, BAC). A diverse sample was sought to ensure varied representation in terms of gender, socioeconomic background and educational level.

### Procedure

The schools were contacted to schedule a meeting with the head teachers. A follow-up meeting was then held with the physical education department to explain the study procedures and agree on the dates for administering the questionnaire to the students. The data collection process took place between March 2022 and May 2023. In a controlled environment, students were given the printed SCQ-S questionnaire consisting of 27 questions, to which 2 control questions were added. The control questions corresponded to the reverse of item 1 (located at position 10) and the reverse of item 10 (located at position 21). Before completing the questionnaire, participants were given instructions, emphasising the importance of anonymity, reflection in responses and honesty. All participants were informed about the objectives of the study and gave their informed consent for the use of the data for scientific purposes. Parental consent was obtained for participants under 16. The study design complied with the ethical principles of the Declaration of Helsinki and was approved by the ethics committee of the University of Alicante under the code UA-2022-03-17. Participation in the study was voluntary and did not involve remuneration.

### Instruments

To achieve the proposed objective, the validated Sustainability Awareness Questionnaire was used, in the abbreviated version (SCQ-S) developed by Gericke et al. [34] and recently validated in Spanish by Boned-Gómez et al. [35]. This instrument was selected for its psychometric robustness and suitability for the adolescent population (RMSEA = 0.041; CFI = 0.953; TLI = 0.944) and for being structured along the dimensions of sustainable development and sustainability awareness. It consists of 27 items distributed across nine subscales, each corresponding to a dimension of sustainability (environmental, social, economic), including three psychological constructs for each associated with sustainability awareness: sustainable knowledge (SK), sustainable attitudes (SA) and sustainable behaviour (SB). This study will focus solely on these six main constructs. The use of the abbreviated version offers the advantage of having fewer questions, which increases the likelihood of participants maintaining their attention and focus while answering the questionnaire. Environmental awareness is understood as a multidimensional function that encompasses cognitive, affective and evaluative aspects. Responses were recorded on a five-point Likert scale, ranging from “strongly disagree” [1] to “strongly agree” [5]. In addition to being closely aligned with the UNESCO framework, this instrument is also related to the 17 most recent SDGs established in the UN’s Global Action Programme [34].

### Statistical analysis

The SPSS 28.0 statistical software was utilised for all analyses. Descriptive statistics for each factor (mean, median, standard deviation and interquartile range) were calculated. The Kolmogorov-Smirnov test for normality was conducted, revealing non-normal distributions in all cases (*p* <.05).

To analyse differences between males and females, the Mann-Whitney U test was applied. A 95% confidence interval for the differences was calculated, and the significance threshold was set at *p* <.05. Additionally, Cronbach’s alpha was employed to assess the questionnaire’s reliability, with results being acceptable (α > 0.70) for all variables except one: Sustainable knowledge (α = 0.785); Sustainable attitude (α = 0.718); Sustainable behaviour (α = 0.768); Environmental dimension (α = 0.608); Social dimension (α = 0.756); and Economic dimension (α = 0.728). Using Microsoft Excel software, the effect size was calculated according to Dominguez-Lara [36]. Finally, the effect size outcomes were categorised into small (0.1–0.3), medium (0.3–0.5) and large (> 0.5) results, following Coolican [37].

## Results

General characteristics of entire sample (males and females; *N* = 1,192) are presented in Table 1.

**Table 1:** Descriptive statistics of the entire sample

	Average	Standard deviation	Min.	Max.	Percentiles		
					25	Median 50	75
SK	4.20	0.62	1	5	3.88	4.33	4.66
SA	4.17	0.55	1	5	3.88	4.33	4.55
SB	3.71	0.72	1	5	3.22	3.77	4.22
EnD	3.85	0.58	1	5	3.44	3.88	4.33
SD	4.26	0.58	1	5	4.00	4.44	4.66
EcD	3.96	0.61	1	5	3.55	4.00	4.44
*SK* Sustainable knowledge, *SA* Sustainable attitude, *SB* Sustainable behaviour, *EnD* Environmental dimension, *SD* Social dimension, *EcD* Economic dimension							

**Fig. 1:**
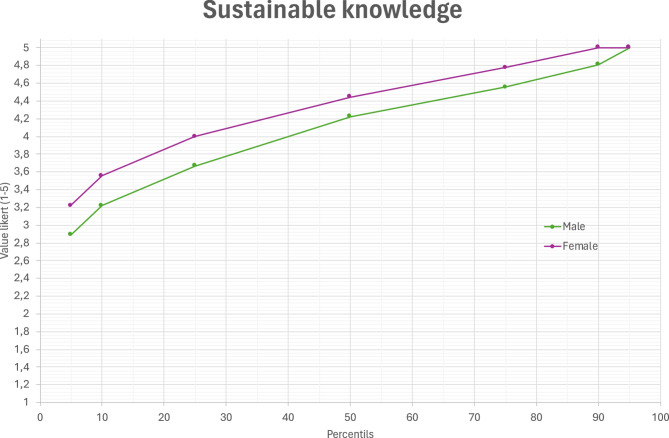
Line graph representative of the sustainable knowledge variable (the rest of the variables are the same pattern)

Table 2 shows a comparative analysis between groups (males vs. females) of the group representative values (in this non-parametric distribution, median and interquartile range) in all variables for males and females. Females can be seen to score statistically higher values than males in all knowledge-related variables (see Fig. 1), attitudes and behaviours in sustainability (*p* <.001), as well as in their various dimensions (environmental, social and economic), with a Cohen’s d small effect size (0.17 < ES < 0.23).

**Table 2:** Comparing variables between males (*n* = 576) and females (*n* = 616) using Mann-Whitney test (Median ± Interquartile range)

Variable	Males			Females			Z	Sig.	ES
SK	4.22	±	0.89	4.44	±	0.78	7.208	< 0.001	0.21
SA	4.11	±	0.67	4.33	±	0.56	7.144	< 0.001	0.21
SB	3.66	±	1.00	3.94	±	0.89	6.452	< 0.001	0.19
EnD	3.78	±	0.89	4.00	±	0.67	6.023	< 0.001	0.17
SD	4.22	±	0.78	4.56	±	0.56	8.115	< 0.001	0.23
EcD	3.89	±	0.78	4.22	±	0.78	7.761	< 0.001	0.22
*Sig P-*value, *ES* Effect size, *SK* Sustainable knowledge *SA* Sustainable attitude, *SB* Sustainable behaviour, *EnD* Environmental dimension *SD* Social dimension *EcD* Economic dimension									

If the results are analysed statistically by segmenting the data set according to the academic course (Table 3), the most remarkable finding is that the same previous pattern (females score higher than males) is presented in all courses except for 7th grade or 1 st ESO (no statistically significant differences) and 2nd BAC for the variables SK, SD and EcD. In all cases, the median is bigger in females. To establish benchmark scores, Table 4 presents percentiles to measure levels of sustainability awareness in adolescents.

**Table 3:** Comparing variables between males (*n* = 576) and females (*n* = 6 16) using Mann-Whitney test (Median ± Interquartile range)

Variable	Males			Females			Z	Sig.	ES
7th grade (1st ESO; *n* = 187)									
SK	4.39	±	1.00	4.44	±	1.22	0.243	0.808	-
SA	4.33	±	0.64	4.33	±	0.56	0.015	0.988	-
SB	3.94	±	1.08	4.11	±	0.89	0.919	0.358	-
EnD	4.06	±	0.89	4.22	±	0.78	0.141	0.888	-
SD	4.50	±	0.86	4.56	±	0.67	1.014	0.310	-
EcD	4.11	±	1.00	4.11	±	0.89	0.164	0.870	-
8th grade (2nd ESO; *n* = 190)									
SK	4.22	±	0.75	4.44	±	0.67	3.408	< 0.001	0.25
SA	4.11	±	0.67	4.33	±	0.56	2.772	0.006	0.20
SB	3.61	±	1.08	4.06	±	0.92	3.277	0.001	0.24
EnD	3.89	±	0.86	4.11	±	0.56	2.862	0.004	0.21
SD	4.22	±	0.78	4.56	±	0.56	3.920	< 0.001	0.28
EcD	4.00	±	0.78	4.22	±	0.67	3.461	< 0.001	0.25
9th grade (3rd ESO; *n* = 351)									
SK	4.11	±	1.00	4.33	±	0.78	4.221	< 0.001	0.23
SA	4.11	±	0.67	4.39	±	0.53	5.035	< 0.001	0.27
SB	3.56	±	1.00	3.78	±	1.00	3.923	< 0.001	0.21
EnD	3.67	±	0.89	4.00	±	0.78	3.365	< 0.001	0.18
SD	4.22	±	0.78	4.50	±	0.56	4.728	< 0.001	0.25
EcD	3.78	±	0.89	4.11	±	0.67	5.636	< 0.001	0.30
10th grade (4th ESO; *n* = 241)									
SK	4.11	±	0.69	4.44	±	0.56	5.447	< 0.001	0.35
SA	4.11	±	0.67	4.44	±	0.44	4.331	< 0.001	0.28
SB	3.56	±	1.11	3.78	±	1.00	3.208	0.001	0.21
EnD	3.67	±	0.78	4.00	±	0.78	4.253	< 0.001	0.27
SD	4.11	±	0.78	4.44	±	0.67	5.351	< 0.001	0.34
EcD	3.89	±	0.69	4.11	±	0.67	4.160	< 0.001	0.27
11th grade (1st BAC; *n* = 157)									
SK	4.33	±	0.89	4.44	±	0.67	2.764	0.006	0.22
SA	4.22	±	0.64	4.44	±	0.44	2.733	0.006	0.22
SB	3.67	±	0.97	4.00	±	0.94	2.336	0.019	0.19
EnD	3.72	±	1.00	4.00	±	0.72	2.328	0.020	0.19
SD	4.33	±	0.75	4.56	±	0.44	2.874	0.004	0.23
EcD	3.89	±	0.67	4.33	±	0.67	3.227	0.001	0.26
12th grade (2nd BAC; *n* = 66)									
SK	4.33	±	0.78	4.44	±	0.67	0.871	0.383	-
SA	4.22	±	0.56	4.33	±	0.22	2.061	0.039	0.25
SB	3.67	±	1.00	4.11	±	0.78	2.184	0.029	0.27
EnD	3.78	±	0.67	4.11	±	0.56	2.566	0.010	0.32
SD	4.33	±	0.67	4.44	±	0.56	1.136	0.256	-
EcD	3.89	±	0.67	4.00	±	0.89	1.391	0.164	-
*Sig P-*value, *ES* Effect size, *SK* Sustainable knowledge, *SA* Sustainable attitude, *SB* Sustainable behaviour, *EnD* Environmental dimension, *SD* Social dimension, *EcD* Economic dimension									

**Table 4:** Percentiles of all variables (course*gender)

Grade	Variable	Gender	Percentile						
			5	10	25	50	75	90	95
1 st ESO	SK	Male	2,89	3,22	3,78	4,39	4,78	5,00	5,00
		Female	2,84	3,11	3,67	4,44	4,89	5,00	5,00
	SA	Male	2,98	3,27	3,92	4,33	4,56	4,92	5,00
		Female	2,96	3,18	4,00	4,33	4,56	4,78	5,00
	SB	Male	2,76	3,00	3,36	3,94	4,44	4,92	5,00
		Female	2,56	2,82	3,56	4,11	4,44	4,78	5,00
	EnD	Male	2,98	3,30	3,56	4,06	4,44	4,78	5,00
		Female	2,84	3,11	3,67	4,22	4,44	4,56	4,82
	SD	Male	3,00	3,11	3,92	4,50	4,78	5,00	5,00
		Female	3,00	3,56	4,11	4,56	4,78	5,00	5,00
	EcD	Male	2,98	3,08	3,56	4,11	4,56	4,89	5,00
		Female	2,76	3,02	3,67	4,11	4,56	4,78	5,00
2nd ESO	SK	Male	2,94	3,11	3,81	4,22	4,56	4,78	4,95
		Female	3,44	3,67	4,11	4,44	4,78	5,00	5,00
	SA	Male	3,00	3,00	3,78	4,11	4,44	4,69	5,00
		Female	3,26	3,56	4,00	4,33	4,56	4,74	4,98
	SB	Male	2,56	2,77	3,22	3,61	4,31	4,78	4,90
		Female	3,00	3,14	3,64	4,06	4,56	4,78	4,89
	EnD	Male	2,83	3,00	3,44	3,89	4,31	4,67	4,78
		Female	3,13	3,44	3,89	4,11	4,44	4,67	4,76
	SD	Male	3,00	3,10	3,78	4,22	4,56	4,79	4,95
		Female	3,38	3,78	4,22	4,56	4,78	4,89	5,00
	EcD	Male	2,83	3,00	3,56	4,00	4,33	4,67	4,84
		Female	3,02	3,44	3,89	4,22	4,56	4,74	4,87
3rd ESO	SK	Male	2,73	3,00	3,56	4,11	4,56	4,78	4,89
		Female	3,22	3,44	4,00	4,33	4,78	4,89	5,00
	SA	Male	2,96	3,02	3,78	4,11	4,44	4,67	4,89
		Female	3,45	3,78	4,03	4,39	4,56	4,88	4,89
	SB	Male	2,22	2,47	3,00	3,56	4,00	4,31	4,56
		Female	2,23	2,57	3,33	3,78	4,33	4,56	4,78
	EnD	Male	2,62	2,89	3,22	3,67	4,11	4,44	4,56
		Female	2,78	3,12	3,56	4,00	4,33	4,56	4,67
	SD	Male	2,96	3,22	3,78	4,22	4,56	4,78	4,89
		Female	3,44	3,68	4,11	4,50	4,67	4,89	5,00
	EcD	Male	2,67	2,89	3,22	3,78	4,11	4,56	4,56
		Female	3,11	3,33	3,78	4,11	4,44	4,78	4,89
4th ESO	SK	Male	2,95	3,22	3,64	4,11	4,33	4,56	5,00
		Female	3,51	3,78	4,11	4,44	4,67	5,00	5,00
	SA	Male	3,11	3,33	3,67	4,11	4,33	4,67	4,83
		Female	3,33	3,58	4,11	4,44	4,56	4,78	5,00
	SB	Male	2,33	2,44	2,89	3,56	4,00	4,22	4,61
		Female	2,58	2,80	3,33	3,78	4,33	4,67	4,82
	EnD	Male	2,62	2,89	3,22	3,67	4,00	4,33	4,56
		Female	2,96	3,22	3,56	4,00	4,33	4,56	4,60
	SD	Male	3,00	3,22	3,67	4,11	4,44	4,67	4,78
		Female	3,67	3,89	4,11	4,44	4,78	4,89	5,00
	EcD	Male	2,95	3,11	3,42	3,89	4,11	4,56	4,77
		Female	3,22	3,44	3,78	4,11	4,44	4,78	4,89
1 st BAC	SK	Male	2,94	3,22	3,78	4,33	4,67	4,89	4,95
		Female	3,33	3,78	4,11	4,44	4,78	5,00	5,00
	SA	Male	3,00	3,32	3,81	4,22	4,44	4,56	4,62
		Female	3,11	3,56	4,11	4,44	4,56	4,89	5,00
	SB	Male	2,15	2,44	3,03	3,67	4,00	4,36	4,67
		Female	2,28	2,67	3,39	4,00	4,33	4,67	4,83
	EnD	Male	2,78	2,89	3,22	3,72	4,22	4,46	4,56
		Female	2,78	3,11	3,61	4,00	4,33	4,56	4,83
	SD	Male	2,66	3,21	3,92	4,33	4,67	4,78	4,89
		Female	3,33	3,89	4,33	4,56	4,78	5,00	5,00
	EcD	Male	2,66	3,21	3,56	3,89	4,22	4,57	4,89
		Female	2,94	3,22	3,89	4,33	4,56	4,78	4,94
2nd BAC	SK	Male	3,44	3,49	3,78	4,33	4,56	4,73	4,87
		Female	3,13	3,36	3,89	4,44	4,56	4,89	4,98
	SA	Male	3,58	3,67	3,89	4,22	4,44	4,56	4,76
		Female	3,44	3,93	4,22	4,33	4,44	4,73	4,96
	SB	Male	2,47	2,71	3,11	3,67	4,11	4,51	4,87
		Female	2,47	3,00	3,56	4,11	4,33	4,58	4,76
	EnD	Male	3,11	3,22	3,44	3,78	4,11	4,36	4,64
		Female	3,33	3,33	3,78	4,11	4,33	4,44	4,62
	SD	Male	3,33	3,47	3,89	4,33	4,56	4,78	4,98
		Female	3,20	3,73	4,22	4,44	4,78	4,96	5,00
	EcD	Male	3,13	3,38	3,56	3,89	4,22	4,51	4,87
		Female	3,02	3,11	3,67	4,00	4,56	4,78	4,96

*SK* Sustainable knowledge, *SA* Sustainable attitude, *SB* Sustainable behaviour, *EnD* Environmental dimension, *SD* Social dimension, *EcD* Economic dimension

## Discussion

The aim of this study was to explore gender differences in sustainability awareness among adolescents, identifying how knowledge, attitudes and behaviours related to sustainability vary between males and females. Previous literature has revealed a growing concern for sustainability but has hinted at an insufficiently examined differentiation in the depth with which each gender approaches the social, economic and environmental dimensions of this issue. The results obtained from our research show that adolescent females exhibit a higher awareness of sustainability than their male counterparts across several dimensions, except for one, where no significant gender differences were observed. This increased awareness among females could be related to differences in gender socialisation, where females are encouraged to have greater empathy and concern for collective well-being, which are factors that may influence their perception and attitude regarding sustainability. The findings of this study suggest that sustainability awareness among adolescents is influenced by gender, thereby highlighting the importance of incorporating gender perspectives in the development of educational programmes and intervention strategies that promote sustainable awareness in this population.

This pattern of gender differences in sustainability awareness aligns with the findings of Olsson and Gericke [22], who identified a gender gap in sustainability awareness among Swedish students, showing an increase in this gap across age ranges, and with it being wider in schools oriented towards ESD. It would also support Lee’s [30] findings that girls were more concerned about the environment. Although they contradict the results of Calabrese et al. [31], this may be because the age range of their sample was higher than that of the present study. In addition to these explanations, other ideas have been proposed in the previous literature. One possible explanation for women’s greater inclination towards sustainability lies in the psychological and emotional differences between genders. Women tend to exhibit higher levels of empathy, which potentially makes them more aware of the repercussions of their actions on the environment and future generations [38]. This empathy, combined with socialisation that emphasises caregiving, concern for others, and an orientation towards collective well-being, could closely align women with the principles of sustainability [39]. Moreover, it is argued that women and men possess distinct sustainability values, which could explain the differences in awareness and sustainable behaviour. Women, characterised by greater empathy towards collective well-being and higher emotional intelligence, might be better equipped to understand and connect with social, economic and environmental challenges at a deeper level [40]. This ability to emotionally relate to issues may reinforce the relationship between mindfulness and sustainability awareness, potentially leading to a greater commitment to sustainable practices [41]. Another aspect to consider is how gender differences in sustainability may be influenced by structural and role factors rather than inherent differences between men and women. For example, variations in sustainable mobility between genders could reflect differences in daily tasks and access to resources, rather than an innate disposition towards sustainability [42].

This perspective suggests that social structures and role expectations can play a significant role in how individuals of different genders relate to sustainability. Although our results are clear regarding the acceptance of the hypothesis, the debate on the impact of gender on sustainability is mixed and often contradictory. On the one hand, recent research suggests that there are no significant differences between men and women in terms of sustainable consumption behaviours, which raises questions about the actual influence of gender in this area [43]. Conversely, other studies suggest that men may exhibit greater knowledge of sustainability issues than women, which challenges the notion that environmental awareness is higher among females [44]. However, this view is countered by research that positions women as more committed and optimistic towards environmental issues than their male counterparts [45]. Therefore, the convergence of these factors highlights the complexity of analysing sustainability awareness through the lens of gender. Although the research presents varied findings, the accumulation of evidence suggests a trend towards greater awareness and commitment of women towards sustainability, driven by psychological, emotional and possibly structural differences. Recognising and understanding these differences is crucial for developing sustainability strategies that are inclusive and effective, capitalising on the strengths and addressing the specific needs of both genders.

The analysis segmented by academic year in our study reveals a consistent pattern of higher scores in sustainability awareness among women compared to men, with notable exceptions at certain educational levels. This finding underscores the complexity of the influence that the educational context and developmental evolution have on the formation of sustainability awareness among adolescents. It is evident that educational interventions in the field of sustainability can have varied effects depending on the educational level and other contextual factors yet to be explored. Olsson, Gericke and Chang Rundgren [28] indicate that awareness of sustainability tends to decrease during adolescence, which suggests the need to tailor ESD to this specific age group. This phenomenon, known as “the adolescent decline” in sustainability awareness, implies that educational strategies must be carefully designed to be effective at each stage of a student’s educational and cognitive development. In this regard, Eccles and Roeser [46] highlight the importance of considering the school context as a crucial developmental environment for adolescents, suggesting that school characteristics, such as teaching practices and school climate, play a significant role in the intellectual and socioemotional development of students. This may reinforce the idea that the implementation of ESD must consider how these school factors can facilitate or hinder the adoption of sustainable attitudes and behaviours [47]. Moreover, Wals and Jickling [48] argue the importance of higher education, which has the responsibility to challenge and question the value and knowledge claims that have prescriptive tendencies, particularly in regard to sustainability. They propose that engaging students in socioscientific disputes about sustainability can enhance the quality of the learning process, making sustainability not just a subject of study but also a critical framework for thinking and meaningful learning.

Detecting this gender gap is also important in order to be able to intervene from adolescence and thus promote equitable gender attitudes that support a sustainable approach in future generations [49]. In this regard, Levy et al. [50] highlight the effectiveness of programmes aimed at transforming restrictive gender norms, emphasising the need for initiatives that focus not only on the individual but also on their interpersonal and community environment – an aspect that could similarly influence the development of a broader sustainable awareness. These findings underscore an underlying complexity in how gender norms and socially constructed roles can influence the perception of and engagement with sustainability issues from an early age. Therefore, we must be aware of the importance of the relationship between gender inequality in society and gender differences in health behaviours among adolescents, as it could be suggested that restrictive gender norms impact not only on awareness of sustainability but also on a wider range of behaviours and attitudes related to health and well-being [51].

## Limitations and future research

Although this study sheds light on the interaction between gender and sustainability awareness, it is not without limitations that should be considered when interpreting its findings. Firstly, having been conducted in a single country, the ability to generalise results to different cultural contexts may be limited. Furthermore, the sample was drawn from schools in a specific region of Spain, which may not be representative of the diversity of all educational contexts, socio-economic backgrounds or cultural attitudes in the country. This regional concentration may limit the generalisability of the results, as other students from different regions may have different levels of exposure to these contents. For this reason, future studies could seek to include more geographically and demographically diverse samples. Moreover, the influence of the educational curriculum on sustainability awareness (a factor not examined in depth in this study) suggests the need for future research that considers education as a potentially significant variable. Although the study’s sample is extensive, comparisons between different countries within the same region could enrich the understanding of how specific cultural and educational dynamics influence sustainability awareness through the lens of gender. Finally, it should be noted that the results could be subject to response bias, as the data were collected using a self-administered questionnaire with Likert-scale items. This is particularly relevant because participants may be inclined to give socially desirable answers. Although the questionnaire was anonymous and completed in a familiar school environment to minimise pressure, the possibility of over-reporting in favour of sustainability cannot be completely ruled out.

From the perspective of future research, it is imperative to address these limitations by extending the analysis to multiple national contexts and evaluating the impact of educational content on the formation of attitudes towards sustainability. Additionally, exploring how individual differences beyond gender, such as age, socioeconomic level and education, interact with sustainable perceptions and behaviours would be valuable. This line of research would not only broaden our understanding of the complexities underlying sustainability awareness but could also identify specific intervention points for educational programmes and public policies. For example, longitudinal studies could be conducted to follow the same cohort of students over several years to investigate how sustainability awareness evolves over time and which factors most affect this development. In addition, experimental research could be conducted to assess the effectiveness of educational interventions aimed at reducing observed gender disparities in sustainability awareness. For example, through school curricula that incorporate emotional intelligence training or collaborative environmental projects.

Regarding practical implications, the findings of this study underline the importance of designing and implementing education and communication strategies in sustainability that are sensitive to gender differences. Educational institutions and policymakers could use these insights to develop curricula and awareness campaigns that recognise and leverage the differences in awareness and motivation towards sustainability between men and women. For example, focusing sustainability education efforts on promoting empathy and emotional intelligence could be particularly effective in increasing environmental awareness among youths. Similarly, adapting sustainability policies and programmes to address and capitalise on the identified gender differences could contribute to more equitable and effective participation in environmental preservation. This approach would not only enrich current sustainability strategies but also promote a more inclusive society that is aware of the unique contributions of each individual towards a sustainable future. These findings highlight the need for gender-sensitive and inclusive sustainability education that ensures equal engagement and interest from all students, regardless of gender identity.

## Conclusions

Working towards equal treatment of genders in education is presented not only as an ethical commitment to social justice but also as a fundamental necessity to foster global SD. Through the SDGs, especially SDGs 4 and 5, we are provided with a clear guide for integrating gender equality into educational systems worldwide. This integration requires the careful design of educational interventions that recognise and address the specific needs of genders, ensuring that both girls and boys have access to equitable educational opportunities that maximise their individual and collective potential for the benefit of SD. The analysis of the results underscores the importance of adapting educational interventions to be inclusive and sensitive to gender differences, which is crucial not only for improving sustainability awareness among students but also for advancing towards a more equitable and sustainable society. This study emphasises the need to adopt a holistic and adaptive approach in teaching sustainability, considering the differences in awareness and sustainable behaviours across various stages of educational development and the significant impact that the school environment can have on this learning. Hence, the effectiveness of any educational intervention in sustainability will depend on a detailed understanding of how gender factors and the educational context interact and affect learning and the adoption of sustainable behaviours. This understanding is vital for the development of policies and educational strategies that not only aim to achieve gender equality but also promote active and conscious participation in the SD of communities and the planet. In conclusion, addressing gender disparities in ESD is a critical step towards building fairer, healthier and more resilient societies.

## Data Availability

Data is available upon request.

## Abbreviations

SD: sustainable development
ESD: education for sustainable development
SDGs: Sustainable Development Goals
STEM: science, technology, engineering and mathematics
ESO: compulsory secondary education
BAC: Bachillerato (non-compulsory secondary education)
SCQ-S: Sustainability Awareness Questionnaire abbreviated version
SK: Sustainable knowledge
SA: Sustainable attitude
SB: Sustainable behaviour
EnD: Environmental dimension
SD: Social dimension
EcD: Economic dimension
